# Altering Chemosensitivity by Modulating Translation Elongation

**DOI:** 10.1371/journal.pone.0005428

**Published:** 2009-05-01

**Authors:** Francis Robert, Marilyn Carrier, Svea Rawe, Samuel Chen, Scott Lowe, Jerry Pelletier

**Affiliations:** 1 Department of Biochemistry, McGill University, Montreal, Quebec, Canada; 2 Howard Hughes Medical Institute, Cold Spring Harbor Laboratory, Cold Spring Harbor, New York, United States of America; 3 McGill Cancer Center, McGill University, Montreal, Quebec, Canada; University of Edinburgh, United Kingdom

## Abstract

**Background:**

The process of translation occurs at a nexus point downstream of a number of signal pathways and developmental processes. Modeling activation of the PTEN/AKT/mTOR pathway in the Eμ-Myc mouse is a valuable tool to study tumor genotype/chemosensitivity relationships *in vivo*. In this model, blocking translation initiation with silvestrol, an inhibitor of the ribosome recruitment step has been showed to modulate the sensitivity of the tumors to the effect of standard chemotherapy. However, inhibitors of translation elongation have been tested as potential anti-cancer therapeutic agents *in vitro*, but have not been extensively tested in genetically well-defined mouse tumor models or for potential synergy with standard of care agents.

**Methodology/Principal Findings:**

Here, we chose four structurally different chemical inhibitors of translation elongation: homoharringtonine, bruceantin, didemnin B and cycloheximide, and tested their ability to alter the chemoresistance of Eμ-myc lymphomas harbouring lesions in *Pten*, *Tsc2*, *Bcl-2*, or *eIF4E*. We show that in some genetic settings, translation elongation inhibitors are able to synergize with doxorubicin by reinstating an apoptotic program in tumor cells. We attribute this effect to a reduction in levels of pro-oncogenic or pro-survival proteins having short half-lives, like Mcl-1, cyclin D1 or c-Myc. Using lymphomas cells grown *ex vivo* we reproduced the synergy observed in mice between chemotherapy and elongation inhibition and show that this is reversed by blocking protein degradation with a proteasome inhibitor.

**Conclusion/Significance:**

Our results indicate that depleting short-lived pro-survival factors by inhibiting their synthesis could achieve a therapeutic response in tumors harboring PTEN/AKT/mTOR pathway mutations.

## Introduction

Recent large scale analysis of gene mutations, deletions, and amplifications in human tumors have revealed that cancers exhibit on average ∼60–90 genetic alterations per tumor [1], [2]. The majority of these genetic alterations target players in a limited set of signalling transduction pathways or processes [1], [2]. These analyses suggest that therapeutic targeting of specific altered oncogenes may be too narrow an approach for drug development, but rather targeting nodes that reside downstream of these pathways may offer broader acting therapies [1], [2]. Indeed, the process of translation is a node for several signalling pathways and has been shown to be a potential therapeutic target.

One approach to study genotype-drug response relationships has been the use of mechanism-based mouse cancer models, such as the Eμ-Myc lymphoma model [3]. In this model, activating lesions in PI3K/AKT/mTOR signalling not only accelerate tumorigenesis, but also modulate chemosensitivity [4], [5], [6]. Resistance to doxorubicin (Dxr) or cyclophosphamide in myr-AKT activated or *PTEN^+/−^Eμ-Myc* lymphomas has been linked to (a) defective apoptotic program(s) dependent on increased mTOR activity and linked to elevated translation initiation rates.

mTOR impinges on the translation process by regulating the assembly of eukaryotic initiation factor (eIF) 4F, a heterotrimeric complex consisting of: eIF4E, a cap (m^7^GpppN, where N in any nucleotide) binding protein; eIF4A, a DEAD-box RNA helicase; and eIF4G, a large scaffolding protein involved in recruiting the 40S ribosome (and associated factors) [7]. Increased eIF4F activity is thought to increase translation rates since eIF4E is the least abundant translation factor and initiation is generally rate-limiting for translation [8], [9]. Increased eIF4F activity stimulates preferentially the translation of mRNA with G+C rich, highly-structured 5′UTRs (weak mRNAs) without significantly affecting translation of mRNAs with short and unstructured 5′UTRs (strong mRNAs) [10], [11], [12]. Typically, strong mRNAs encode house keeping genes like β-actin and GAPDH whereas weak mRNAs encode potent growth and survival factors, such as the angiogenesis factors VEGF and FGF-2, the proto-oncoproteins cyclin D1 and c-Myc, and the pro-survival factors myeloid cell leukemia sequence 1 (Mcl-1) and survivin [13], [14], [15].

Preventing eIF4F assembly by inhibiting mTOR signalling with rapamycin (Rap) [6], [16] or blocking eIF4F activity with silvestrol, an inhibitor of the ribosome-recruitment step of translation initiation [17], can sensitize Eμ-Myc lymphomas with elevated mTOR signalling to the cytotoxic action of Dxr, though neither of these treatments on their own is effective. eIF4E is oncogenic *in vivo* and in Eμ-Myc lymphomas over-expressing this factor, rapamycin is unable to modulate chemosensitivity whereas silvestrol can [6], [16], [18], [19]. The mechanism by which Rap and silvestrol alter chemosensitivity is not clear but may involve remodelling of the oncoproteome through differential mRNA recruitment into initiation complexes [13], with preferred inhibition of “weak” mRNAs encoding pro-survival signals, such as Mcl-1 [4], [17], [20]

Interestingly, several inhibitors of translation elongation have also been reported to exert significant anti-cancer activity. This creates somewhat of a paradox as elongation inhibitors are not expected to be selective in their mode of action and would be expected to possess a narrow therapeutic window. Yet, homoharringtonine (HHT) (a cephalotaxus alkaloid) has demonstrated activity in patients with chronic myeloid leukemia after imatinib failure [21]. Aplidine [a didemnim (Did) family member] has shown activity in phase I clinical trials for many cancer types but especially in advanced medullar thyroid carcinoma [22]. Bruceantin [(Bru); a quassinoid], showed efficacy in a RPMI 8226 human-SCID xenografts mouse model [23].

To address whether inhibitors of translation elongation could also synergize with DNA damaging agents (e.g. Dxr), we tested the potential of four elongation inhibitors to modulate chemosensitivity in the Eμ-myc model harboring lymphomas with loss of Pten or Tsc2 or over-expressing Bcl-2 or eIF4E. We find that all inhibitors tested can alter the chemosensitivity of tumors harboring activated mTOR, but not in Bcl-2-driven tumors. We observe that these compounds reduce Mcl-1 levels and postulate that they cause a reduction in the levels of short-lived proteins, some of which are pro-survival factors. We propose that this resets the apoptotic program. These results provide a mechanism by which elongation inhibitors sensitize cells to apoptotic triggers.

## Materials and Methods

### Ethics Statement

Animal studies were approved by the McGill University Faculty of Medicine Animal Care Committee.

### Compound preparation and storage

HHT (Sigma-Aldrich, St.-Louis, MO), cycloheximide (CHX) (BioShop, Burlington, On), didemnin B (Did B) (NCI-DTP), Bru (NCI-DTP) and MG132 (Sigma-Aldrich, St.-Louis, MO) were resuspended in DMSO and stored at −70°C. Doxorubicin (Sigma-Aldrich, St.-Louis, MO) was dissolved in water and stored at 4°C. Rap (LC Laboratories, Woburn, MA) was resuspended in 100% ethanol and stored at −70°C.

### Cell culture and *in vitro* synergy assays

Mice bearing palpable *Tsc2^+/−^Eμ-myc* lymphomas were sacrificed, lymph nodes extracted, and tumor cells harvested by gently crushing the lymph nodes between two microscope slides. Cells were then put into culture in B-cell medium (BCM) (50% DMEM/50% IMDM) (Invitrogen, Carlsbad, CA), supplemented with 55 µM β-mercaptoethanol, Pen/Strep (Invitrogen, Carlsbad, CA) and Glutamine (Invitrogen, Carlsbad, CA) over an irradiated NIH3T3 feeder layer. For *ex vivo* synergy experiments, 10^5^ *Tsc2^+/−^Eμ-myc* cells were plated in 96-well plates in the presence of HHT (0.625 nM to 160 nM), MG132 (0.039 µM to 20 µM), Dxr (0.0975 µg/ml to 10 ug/ml) or various combinations thereof. Sixteen hours later, cell viability was assessed with the CellTiter 96® Non-Radioactive Cell Proliferation Assay (MTT) according to the manufacturer recommendations (Promega, Madison, WI). Synergy between compound treatments was calculated using compusyn software (ComboSyn Inc.).

### Treatment studies

The generation of *Pten^+/−^Eμ-Myc*, *Tsc2^+/−^Eμ-Myc*, *Eμ-Myc/Bcl-2* and *Eμ-Myc/eIF4E* lymphomas has been previously described [6], [16]. A total of 10^6^ secondary lymphoma cells were injected into the tail vein of 6–8 week old female C57BL/6 mice. When well-palpable tumors arose, mice were treated with Dxr (once at 10 mg/kg), Rap (4 mg/kg daily for 5 days), HHT (0.25 mg/kg daily for 5 days), Bru (0.5 mg/kg daily for 5 days), Did B (0.05 mg/kg daily for 5 days), or CHX (12.5 mg/kg for 5 days). The compounds were diluted into 5.2% PEG400/5.2% Tween 80 immediately prior to intraperitoneal (IP) injection. In combination studies, Rap, HHT, Bru, Did B or CHX were administered once daily for 5 consecutive days, with Dxr being administered once on day two. The presence of the tumors was monitored by daily palpation and blood smears (twice/week) stained with Hema-3® stain (Fisher Scientific, Pittsburg, PA). Tumor-free survival is defined as the time between tumor disappearance and reappearance of a palpable lymphoma. Overall survival is defined as the time after tumor disappearance required for the mice to reach a terminal stage at which point the animals were sacrificed. The data were analyzed in the Kaplan-Meier format using the log-rank (Mantel-Cox) test for statistical significance (SigmaStat software).

### TUNEL (terminal deoxyribonucleotide transferase-mediate nick-end labeling) assays and Western blotting

For TUNEL assays, 6–8 week old C57Bl/6 mice bearing well-palpable tumors were treated once with Rap (4 mg/kg), HHT (0.25 mg/kg), Bru (0.5 mg/kg), Did B (0.5 mg/kg), or CHX (12.5 mg/kg) and the next day were treated again with or without Dxr (10 mg/kg). Four hours later, tumors were removed and fixed in 10% Neutral Buffered Formalin (NBF) overnight and embedded in paraffin. Tumor sections (4 µm) were used in TUNEL assays according to the manufacturer's recommendations (Roche Applied Science, Indianapolis, IN) and stained with Hematoxylin to visualize cell boundaries. For Western blot analysis, mice were treated for the indicated times, the tumors harvested, total cell lysates prepared using RIPA buffer, and proteins separated by SDS-PAGE. The antibodies used were: β-actin (Sigma-Aldrich, St.-Louis, MI), cleaved PARP (Cell Signalling, Beverly, MA), Mcl-1 (Rockland antibodies, Gilbertsville, PA), cyclin D1 (Cell signalling, Beverly, MA), c-Myc (Santa cruz biotechnology, Santa Cruz, CA), and α-tubulin (Sigma-Aldrich, St.-Louis, MI). Total protein content of each extract was determined using the DC protein assay (Biorad, Richmond, CA).

### Ribosome binding and polysome analysis

Ribosome binding experiments were performed as previously described [24]. In brief, ^32^P-labelled CAT mRNA was incubated with rabbit reticulocyte lysate in the presence of 10 µM HHT+600 µM CHX, 10 µM Bru+600 µM CHX, CHX alone or no compounds for 10 min at 30°C. Translation initiation complexes formed were resolved on 10–30% glycerol gradients by centrifugation for 3.5 h at 39,000 rpm (187,000×g) in an SW41 rotor. The gradients were then fractionated using a UA-6 UV detector (ISCO, Lincoln, NE) with a Brandel tube piercer and the radioactivity in each fraction was determined by Cerenkov counting.

For polysome analysis, mice bearing well-palpable lymphomas were injected IP with HHT (0.25 mg/kg) or Bru (0.5 mg/kg) diluted into 5.2% PEG400/5.2% Tween 80. Two hours later, the tumors were harvested, washed with PBS and lysed in hypotonic lysis buffer (5 mM Tris_7.5_, 2.5 mM MgCl_2_, 1.5 mM KCl, 2 mM DTT, 1% Triton X-100, 0.5% Sodium Deoxycholate) in the presence of 100 µg/mL CHX. The extracts were centrifuged through a 10–50% sucrose gradient at 35,000 rpm (150,000×g) for 2 h in an SW40 rotor, followed by fractionation of the gradients using a UA-6 UV detector (ISCO, Lincoln, NE) using a Brandel tube piercer. Total RNA from every second fraction was isolated using Trizol (Invitrogen Carlsbad, CA). The amount of Mcl-1, Cyclin D1, c-Myc and β-actin mRNAs were detected by qRT-PCR using the Roche Diagnostics LightCycler instrument and LightCycler RNA master SYBR green I kit according to the manufacturer's instruction. The primers used for qRT-PCR were: c-Myc: ^5′^TGCGACTGACCCAACATCAG^3′^ and ^5′^CCTGTCCTGGCTCGCAGATT^3′^ ; Cyclin D1: ^5′^CAGGTTCCTGTTCACAATACCTCA^3′^ and ^5′^AGACCGCCCACCTGCC^3′^ , Mcl-1 ^5′^AGCACATTTCTGATGCCGCCT^3′^ and ^5′^GTGCCTTTGTGGCCAAACACT^3′^ ; and β-actin: ^5′^TCACTATTGGCAACGAGCGGTT^3′^ and ^5′^TGTCAGCAATGCCTGGGTACAT^3′^ .

## Results

### Inhibition of translation elongation can alter chemosensitivity

**Figure 1:**
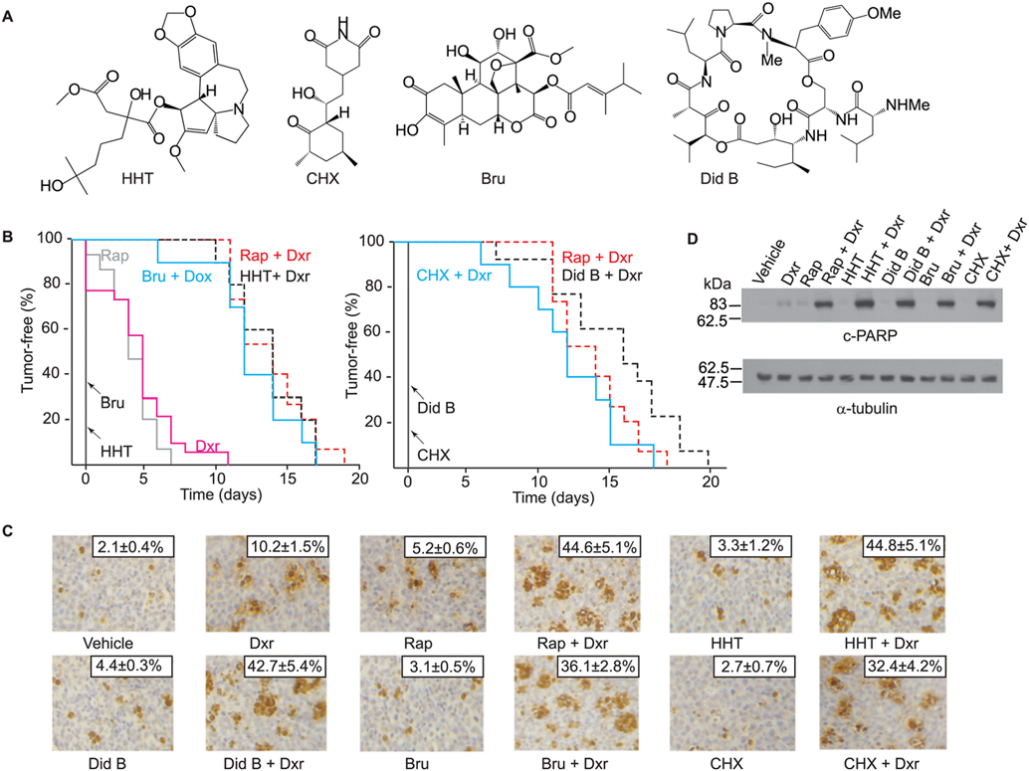
Translation elongation inhibitors alter the chemosensitivity of *Pten^+/−^Eμ-Myc* lymphomas. A. Schematic diagram illustrating the chemical structure of elongation inhibitors used in this study. B. Kaplan-Meier curves representing the time to relapse following treatment of mice bearing *Pten^+−^Eμ-Myc* tumors. Ten animals were treated in each cohort. All mice were treated at the same time and in the same experiment, but the data is presented as two curves for ease of visualization. P<0.001 for significance among all curves of combination treatments compared to single agent treatments, as determined by the log rank test. C. Translation elongation inhibitors potentiate the apoptotic program induced by Dxr in *Pten^+/−^Eμ-Myc* lymphomas *in vivo*. Representative micrographs of *Pten^+/−^Eμ-Myc* lymphomas following treatments (original magnification, ×200). Mice were first treated with elongation inhibitors or Rap and injected again 24 hrs later with the same compounds with or without combination treatment with Dxr. Four hours later, tumors were extracted and processed for TUNEL analysis. The percentage of cells that stained positive is indicated at the top right and represents the average of three different fields where 500 cells were counted per field. This experiment was repeated three times with similar results. D. Representative Western blot analysis of *Pten^+/−^Eμ-Myc* lymphomas treated as described in (C). Tumor cells were extracted, lysed, and the levels of cleaved-PARP (c-PARP) and α-tubulin determined by Western blotting. This experiment was performed on three independent tumor samples with similar results.

A large number of translation elongation inhibitors have been tested in NIH's Developmental Therapeutics Program to assess the chemotherapeutic potential of small molecules in various mouse cancer models as single agents (Fig. S1). A significant fraction of these show activity, although in many cases, these results are restricted to a single dose, delivery route, or cancer model. In addition, a shortcoming of these assays is that they do not assess for a compounds' ability to modulate chemosensitivity. We therefore chose to use the Eμ-Myc lymphoma model to assess the anti-cancer and chemosensitization activity of translation elongation inhibitors. In this study, we used four structurally different elongation inhibitors: HHT, CHX, Bru, and Did B (Fig. 1A). HHT and Bru inhibit the first steps of elongation whereas Did B and CHX block translocation by interfering with elongation factor and E site function, respectively ([25], and this report).

Compounds were tested in combination with Dxr, using a delivery regiment consisting of 5 daily injections of compound, with delivery of one bolus of Dxr on the second day of treatment [16]. For these studies, we used mice bearing *Pten^+/−^Eμ-Myc* lymphomas since mTOR is activated in these tumors and they respond to the combination of Dxr and Rap treatment [16], as well as to combinations of Dxr and the translation initiation inhibitor, silvestrol [17]. As single agents, none of the elongation inhibitors tested induced remissions (Fig. 1B). Dxr and Rap as single agents induced a slight remission that lasted on average 5 days, as previously reported [17]. In contrast, all four elongation inhibitors and Rap synergized with Dxr to extend the tumor-free period and overall survival 2–3 fold (Figs 1B and S2). Enhanced drug sensitivity was associated with increased apoptosis for all drug treatment combinations, compared to single agent treatments (Fig. 1C). Consistent with this interpretation, the observed enhanced sensitivity to Dxr/elongation inhibitor combination was also associated with elevated PARP cleavage (Fig. 1D). As single agents, none of the inhibitors significantly induced PARP cleavage (Fig. 1D).

**Figure 2:**
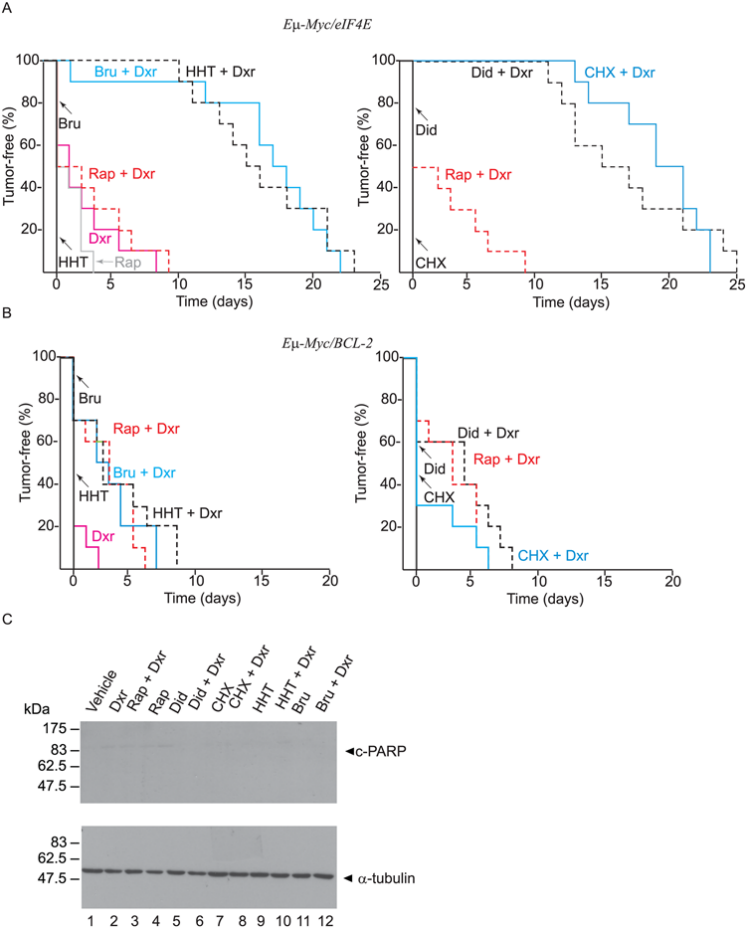
Translation elongation inhibitors alter the chemosensitivity of *Eμ-Myc/eIF4E* lymphomas. A. Kaplan-Meier curves representing the time to relapse following treatment of mice bearing *Eμ-Myc/eIF4E* tumors. Ten animals were treated in each cohort. All mice were treated at the same time and in the same experiment, but the data is presented as two curves for ease of visualization. P<0.001 for significance among all curves of combination treatments involving Bru+Dxr, HHT+Dxr, Did+Dxr, and CHX+Dxr compared to single agent treatments, as determined by the log rank test. B. *Eμ-Myc/Bcl-2* tumors are highly resistant to the combination of translation elongation inhibition and Dxr *in vivo*. Kaplan-Meier plot showing tumor-free survival of mice (n = 10 for each cohort) bearing *Eμ-Myc/Bcl-2* tumors following treatment. Log rank analysis of the curves of combination relative to single agent treatments shows a significant difference between Dxr and Rap+Dxr, HHT+Dxr, Did+Dxr and Bru+Dxr, but not CHX+Dxr with P-values of 0.003, 0.002, 0.004, 0.003 and 0.120, respectively. C. Western blot analysis of *Eμ-Myc/Bcl-2* lymphomas treated as indicated above the panel. Tumor cells were extracted, lysed, and the levels of cleaved-PARP (c-PARP) and α-tubulin determined by Western blotting. The Western blots were performed on two independent tumor samples with similar results.

To assess the efficacy of translation elongation inhibitors on Rap-resistant lymphomas, we examined the activity of these compounds on *Eμ-Myc/eIF4E* lymphomas. These lymphomas over-express eIF4E and are resistant to Dxr+Rap combination treatment [6], [16] (Fig. 2A), but sensitive to the combination of Dxr and silvestrol, an eIF4A activity modulator [17]. As observed with *Pten^+/−^Eμ-Myc* lymphomas, treatment of mice bearing *Eμ-Myc/eIF4E* lymphomas with any of the elongation inhibitors alone was not effective in inducing remissions (Fig. 2A). Treatment with Dxr, Rap, or Rap+Dxr caused a short-lived remission (∼5 days) in ∼50% of the mice (Fig. 2A). However, combination therapy with any one of the four translation inhibitors and Dxr produced tumor-free remissions that lasted up to 25 days (Fig. 2A) and also extended the survival of the mice (Fig. S3A). We do not believe that an inherent feature of *Eμ-Myc* lymphomas make them particularly sensitive to the effects of elongation inhibitor/Dxr combination since *Eμ-Myc/Bcl-2* lymphomas showed significant resistance to these drug combinations (Fig. 2B). Bcl-2 was able to protect against PARP cleavage by these treatments (Fig. 2C). All treatments that extend the tumor-free period in mice bearing *Eμ-Myc/eIF-4E* lymphomas had no effect on overall survival in mice bearing *Eμ-Myc/Bcl-2* tumors (Fig. S3B).

### Degradation of short-lived proteins following exposure to translation elongation inhibitors

Several of the proteins that play important regulatory roles in cancer progression and apoptosis have short half-lives (e.g. cyclin D1 (∼25 min) [26]; c-Myc (∼25 min) [27]; myeloid cell leukemia-1 (Mcl-1) (∼40 min) [28]. The level of these proteins is particularly sensitive to ongoing protein synthesis rates and can be affected by cellular stresses or treatment with translation inhibitors in cell culture. For example, the anti-apoptotic factor Mcl-1, a short-lived Bcl-2 family member essential for normal haematopoiesis and constitutively targeted to the proteasome by the E3 Ligase MULE [29], is rapidly degraded upon UV-irradiation [30] or HHT treatment [31]. c-Myc protein levels have been shown to rapidly decline upon CHX treatment [32]. Since the inhibitors we were using are predicted to affect global translation, we rationalized that one mechanism by which they could be affecting survival is through allowing the rapid loss of short-lived pro-survival factors from cells treated with translation inhibitors to facilitate pro-apoptotic triggering.

**Figure 3:**
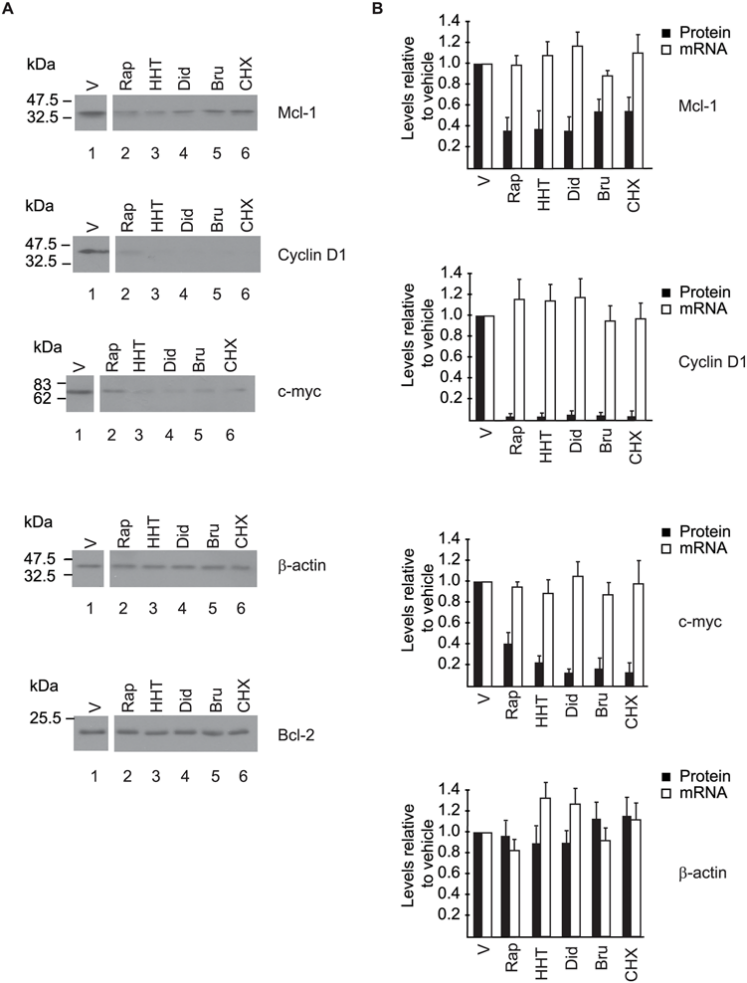
Inhibition of translation elongation in mice bearing *Pten^+/−^Eμ-Myc* lymphomas leads to reduced amounts of Mcl-1, Cyclin D1, and c-Myc. Mice bearing *Pten^+/−^Eμ-Myc* tumors were treated for 28 hrs (two injections; 24 hrs apart) with either Rap (4 mg/kg), HHT (0.25 mg/kg), Bru (0.5 mg/kg), Did B (0.05 mg/kg) or CHX (12.5 mg/kg). A. At 28 hrs (lanes 2–6) tumor cells were extracted, lysed, and proteins fractionated by SDS-PAGE followed by Western blot analysis. The vehicle control mice were treated like the 28 hrs compound treatments except that compounds were omitted. B. Assessment of mRNA levels in *Pten^+/−^Eμ-Myc* tumors by qRT-PCR. The change in mRNA amounts relative to vehicle-treated controls (V) from three independent experiments is shown as a bar graph with standard deviation. Quantitation of the data from three independent experiments performed as in A is also superimposed on these graphs.

We then sought to verify the levels of three short-lived proteins *in vivo* in *Pten^+/−^Eμ-Myc* lymphomas treated in tumor-bearing mice (Fig. 3). Following delivery of 2 doses of compound over a 24-hr period, tumors were isolated 4 hrs after the 2^nd^ injection. By 28 hrs post-drug delivery, there was a reduction in the amount of Mcl-1, Cyclin D1, and c-Myc protein levels in *Pten^+/−^Eμ-Myc* lymphomas relative to vehicle-treated controls (Fig. 3A, compare lanes 2–6 to 1) whereas the levels of β-actin or Bcl-2 did not appreciably change over this time period. Using the same treatment schedule, no significant change in Mcl-1, cyclin D1, and c-Myc mRNA level was observed in the tumors with the elongation inhibitors (Fig 3B).

### HHT and Bru block translation in *Pten^+/−^Eμ-Myc* lymphomas

**Figure 4:**
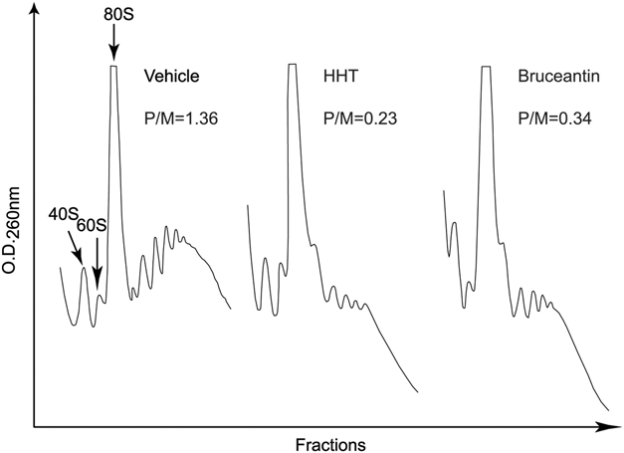
A. HHT and Bru inhibit polysome formation in *Pten^+/−^Eμ-Myc* tumors. Mice bearing well-palpable *Pten^+/−^Eμ-Myc* tumors were treated with either vehicle (DMSO), HHT (0.25 mg/kg), or Bru (0.5 mg/kg). Two hours later, tumors were harvested and cell extracts prepared and fractionated through 10%–50% sucrose gradients. Polyribosomes were monitored by measuring the OD_260_ using an ISCO UA-6 UV detector. The experiment was repeated two more times with similar results.

To provide direct evidence that the compounds being used actually are targeting the translation process in tumor cells *in vivo*; we analyzed the polyribosome content of *Pten^+/−^Eμ-Myc* tumors from mice treated with the elongation inhibitors. We focussed on HHT and Bru, since the polysomes from CHX or Did B treated tumors would resemble untreated controls, as both of these compounds cause ribosome stalling on mRNA templates [25]. Although HHT and Bru are regarded as elongation inhibitors, their mode of action has not been well defined. Both compounds inhibit the peptidyl transferase reaction [33], [34], [35], [36] and are thought to bind tighter to free ribosomes than translating ribosomes [33], [34]. When added to actively translating reactions, there is a 2–4 minute lag before onset of inhibition is observed [33], [37], suggesting that the compounds allow ribosome run-off. To obtain more direct evidence that both these compounds block elongation and do not affect ribosome loading, we tested their ability to counter the formation of the CHX-induced stable 80S complexes on mRNA templates in *in vitro* ribosome binding assays. Sedimentation velocity centrifugation was used to separate 80S complexes from unbound radiolabelled mRNA (Fig. S4). HHT and Bru were thus tested in combination with CHX, to monitor their effect on ribosome loading. The results indicate that, both HHT and Bru do not significantly interfere with the trapping of 80S complexes by CHX when compared to the binding obtained without CHX. Furthermore, when tested as single agents in this assay, HHT and Bru induce a peak comparable in height to the one obtained with CHX alone (data not shown). Taken together with the fact that these compounds allow ribosome run-off, we conclude that they do not affect ribosome loading but rather block the first step of elongation. The polyribosomal content of *Pten^+/−^Eμ-Myc* tumors that had been treated with vehicle, HHT, or Bru indicates a drop in the polyribosomal content of the HHT− and Bru-treated tumors with a concomitant increase in amount of 80S ribosomes (Fig. 4). We found that the polysome to monosome ratio (P/M) in drug-treated tumors decreased ∼4–6 fold compared to vehicle-treated samples (Fig. 4).

### Decreased levels of Mcl-1, Cyclin D1, and c-Myc in *Pten^+/−^Eμ-Myc* tumors treated with translation elongation inhibitors

**Figure 5:**
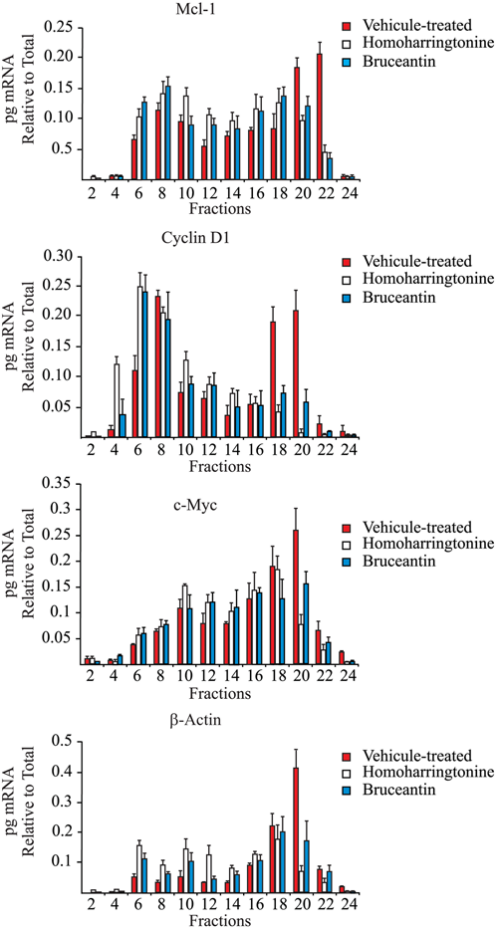
Treatment of mice with HHT and Bru inhibits general protein synthesis *in vivo*. *Pten^+/−^Eμ-Myc* tumors were isolated from HHT− or Bru- treated animals and their polyribosomes fractionated on 10–50% sucrose gradients. RNA was purified from the indicated fractions and quantified using qRT-PCR for Mcl-1, cyclin D1, c-Myc and β-actin levels. The amount of mRNA in each fraction was determined relative to vehicle-treated mice. The values are averaged from three independent experiments and the error bars denote the error of mean (n = 3).

We next addressed whether the decrease in Mcl-1, Cyclin D1, and c-Myc observed upon treatment of *Pten^+/−^Eμ-Myc* tumors with elongation inhibitor was due to a reduction in their translation. To this end, we isolated RNA from fractions that spanned the polyribosomes of *Pten^+/−^Eμ-Myc* tumors taken 2 hr after treatment with HHT−, Bru- or vehicle (Fig. 4). The relative amount of mRNA in every second fraction was determined by qRT-PCR and plotted as a percentage of total mRNA (Fig. 5). Treatment of mice bearing *Pten^+/−^Eμ-Myc* tumors with either HHT or Bru led to a shift in Mcl-1, cyclin D1, c-Myc, and β-actin mRNAs from heavy polysomes (fractions 20–24) into lighter polysomes (fractions 6–12), consistent with these compounds partially inhibiting global protein synthesis *in vivo*. The reasons for the partial inhibition of protein synthesis will be addressed in the Discussion.

**Figure 6:**
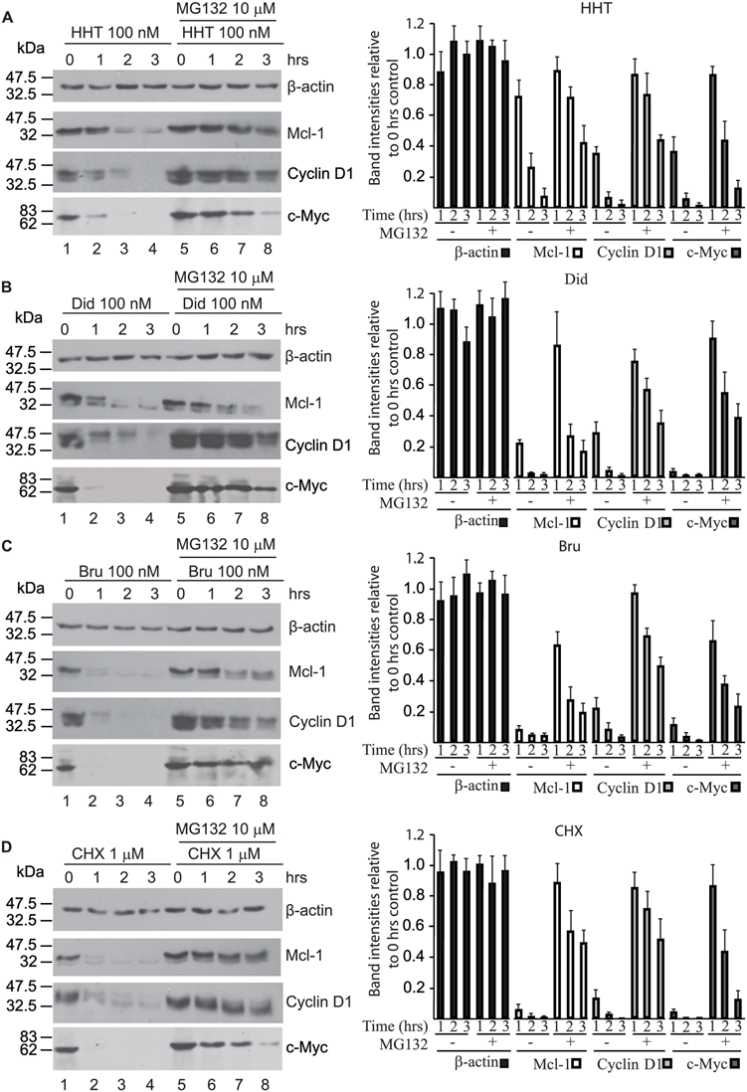
MG132 antagonizes the effects of elongation inhibitors on the levels of Mcl-1, Cyclin D1, and c-Myc. *Tsc2^+/−^Eμ-Myc* lymphomas were pre-treated with MG132 (lanes 5–8) or vehicle (lanes 1–4) for 30 min and exposed to HHT (100 nM) (A), Did B (100 nM) (B), Bru (100 nM) (C) or CHX (1 µM) (D) for the indicated periods of time (lanes 1–8). At the end of each incubation, the cells were harvested, lysed and the levels of Mcl-1, cyclin D1, c-Myc and β-actin determined by Western blotting. The right panel of each gel shows the intensities of each band relative to the 0 hr control with standard deviation from three independent experiments.

**Figure 7:**
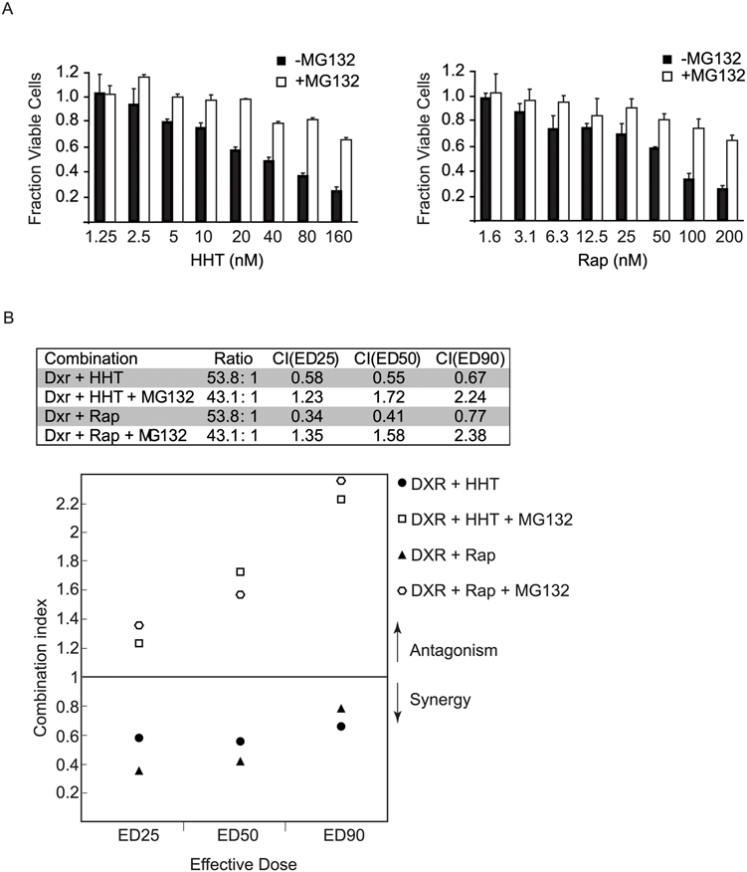
MG132 antagonizes the toxicity of HHT and Rap on *Tsc2^+/−^Eμ-Myc* lymphomas and interferes with their Dxr-associated synergism. A. Cells were exposed to the indicated concentrations of HHT or Rap in the presence or absence of 10 µM MG132. Cell viability was determined 16 hrs later. The results are presented as the fraction of viable cells relative to vehicle (0.02% DMSO) treated samples with the standard deviation presented (n = 3). B. Lymphomas cells were treated with increasing concentrations and HHT and Dxr or Rap and Dxr at a fixed ratio in presence or absence of a fixed MG132 concentration (10 µM) for 16 hrs and the cell viability was determined. A CI value below 1 indicates a synergistic effect whereas a value above 1 represents antagonism. These experiments were performed three times with similar results.

If a reduction in levels of essential short-lived proteins is associated with the synergistic effects observed between inhibitors of elongation and Dxr, then one might expect that the presence of a proteasome inhibitor in this system might antagonize the effects of protein synthesis blockade. For this purpose, we used *Tsc2^+/−^Eμ-Myc* lymphomas cultured *ex vivo* [20]. As documented for *Pten^+/−^Eμ-Myc* lymphomas, inhibiting translation elongation in *Tsc2^+/−^Eμ-Myc* lymphomas also synergizes with Dxr to induce remissions in mice that lasted for up to 28 days (Fig. S5A). Rap also synergized with Dxr in this setting as previously established [4] and was able to inhibit mTOR activity as determined by p-S6 blotting (Fig. S5B). As well, HHT is able to block translation in mice bearing *Tsc2^+/−^Eμ-Myc* lymphomas as revealed by polysome analysis of tumors two hours following compound administration (Fig. S5C). *Tsc2^+/−^Eμ-Myc* lymphomas were cultured *ex vivo* and exposed for various times to a concentration of compound sufficient to completely block global protein synthesis (Fig. S6). Treatments also included a 30-min pre-exposure to the proteasome-inhibitor MG132 [38]. Following incubation, the levels of Mcl-1, c-Myc, Cyclin D1 and β-actin were determined by Western blotting (Fig. 6A–D). As predicted, exposure of cells to HHT, Did B, Bru or CHX lead to a drastic reduction in Mcl-1, cyclin D1, and c-Myc protein levels (compare lanes 2–4 to lane 1). Consistent with this being a selective loss of proteins, β-actin levels did not significantly change (Fig. 6A–D, compare lanes 2–4) nor was the Coomassie-visible protein profile altered (data not shown) when comparing pre- and post-drug exposure. Pre-exposure of cells to MG132 followed by addition of elongation inhibitors strongly blocked the reduction in Mcl-1, Cyclin D1, and c-Myc protein levels (Fig. 6A–D, compare lanes 6–8 to 2–4). Given this result, we sought to determine what effect MG132 would have on HHT− or Rap-induced cell death in *Tsc2^+/−^Eμ-Myc* lymphomas. Tumor cells were exposed to HHT or Rap in the presence or absence of a fixed concentration of MG132 (10 µM) and cell viability determined (Fig 7A). The results indicate that inhibition of the proteasome antagonizes the toxicity of HHT and Rap on these cells with at least 2–3 fold more cells surviving at the highest dose of translation inhibition tested. These results are in agreement with previous work that showed that MG132 can protect against CHX-induced apoptosis in U397 cells [39]. To assess if MG132 would also antagonize the synergy we observed between elongation inhibitors and Dxr, we exposed *Tsc2^+/−^Eμ-myc* lymphomas to HHT or Rap with Dxr. Median effect analysis indicated a combination index (CI) below 1 for Dxr+HHT and Rap+Dxr indicative of synergy [40] (Fig 7B). The presence of MG132 antagonized both Dxr+HHT and Dxr+Rap combinations, with CI values extending above 1 (Fig 7B). The ability of the proteasome inhibitor MG132 to curtail the synergy between HHT and Dxr is consistent with HHT modulating drug sensitivity by causing loss of short-lived pro-survival factors.

## Discussion

Inhibitors of elongation have been previously tested in murine cancer models for their anti-cancer properties as single agents (Fig. S1), as well as identified as genotype-selective antitumor agents in synthetic lethal screens [41]. The potential synergy between translation elongation inhibitors and DNA damaging agents has only been tested in cell culture. CHX has been reported to enhance the cytotoxicity of the anthracycline, epirubicin, against P388 murine leukemic cells [42] and to potentiate Dxr toxicity in RKO-E6 cells [43]. Sparsomycin, pretazettine, and HHT also show synergy with antitumor agents against tumor cells *ex vivo* [44], [45], [46], [47]. Finally, translation elongation inhibitors have been shown to sensitize PC3 cells to TRAIL-induced apoptosis [48] and A549 lung cancer or K562 leukemia cells to cisplatin [49]. In other settings however, CHX and anguidine have protected cells against the cytotoxic effects of antitumor drugs [50], [51], [52]. Differences in underlying genetic lesions among tumors could be responsible for these different responses [53]. Despite the large amount of studies documenting the behaviour of translation elongation inhibitors in cell lines, testing the potential of elongation inhibitors to synergize with standard-of-care agents in animal models has not been systematically approached.

We demonstrate here that the elongation inhibitors HHT, Bru, Did B and CHX modulate the chemosensitivity of *Pten^+/−^Eμ-Myc*, *Tsc2^+/−^Eμ-Myc* and *Eμ-Myc/eIF4E* tumors to the effects of Dxr whereas *Eμ-Myc/Bcl2* tumors were largely refractory to combination therapy. These treatments in the *Pten^+/−^Eμ-Myc* induce an apoptotic response in these chemoresistant tumors as determined by TUNEL analysis and PARP cleavage (Fig. 1). The mechanism by which inhibitors modulate chemoresponsiveness remains to be clearly established, but at the doses tested we observed a correlation between tumor disappearance and inhibition of translation in the tumors (Fig 4A). However, we note that only partial inhibition of protein synthesis was observed *in vivo* with these compounds (Figs. 4A and 5). Pharmacological properties of some of these compounds (serum binding, serum half-life, clearance, or cell permeability) may be the underlying reason why some inhibitors function better than others. The terminal half-life of HHT has been determined to be 14.4 hrs in human [54] and 40.6 hrs in dogs [55]. The clearance of Did B from the blood of human patients is biphasic with apparent half-lives of 0.12 and 4.8 hrs [56] and its observed terminal half-life in mice is 16.2 hrs [57]. The elimination half-life of Bru in mice has been estimated to be greater than 12 hrs and in human serum it is biphasic and progresses through an initial fast half-life of 15 min followed by a second half-life of 0.7–13.8 hrs depending on the patients [58]. Clearly, these compounds display a wide range of pharmacokinetic and pharmacodynamic behaviour.

Our results suggest that a decrease in the levels of factors, like Mcl-1, cyclin D1, and c-Myc might reorganize the onco-proteome allowing transformed cells to become sensitive to Dxr through mechanisms that include re-establishment of the apoptotic program (Fig. 1C). The role of Mcl-1 in enhancing cell survival by blocking activation of the pro-apoptotic factors Bak and Bax has been well-established in culture [39], [59], [60] and c-Myc controls the expression of genes involved in many aspects of cell growth including cell cycle progression and survival [61]. Cyclin D1 has been shown to bind to CDK4 and CDK6 leading to inactivation of Rb1, thus facilitating the transition from G1 to S phase and overcoming the transition inhibition by cyclin dependent kinase inhibitors [62], [63]. It is noteworthy that as single agents, none of these compounds showed activity in the Eμ-myc model, compared to other mouse models where activity was sometimes detected (Fig. S1). Similar results have been documented for the mTOR inhibitor, rapamycin [5], [6], and the eIF4A modulator, silvestrol [17], and suggests that the tumors derived from the Eμ-Myc model require a DNA damage trigger to undergo apoptosis when translation is inhibited.

Many possiblities could explain the resistance of the *Eμ-Myc/Bcl-2* tumors to Dxr/elongation inhibitor combination (Fig. 2B). This could be due to the longer half-life of this anti-apoptotic factor (∼10 hrs) [64] combined to the only partial inhibition of protein synthesis achieved *in vivo* by these compounds (Fig. 5). Alternatively, reduced levels of short-lived proteins (e.g. Mcl-1, cyclin D1, or c-Myc) could be better tolerated in the *Eμ-Myc*/*Bcl-2* tumors than in the other models. Further work would be required to formally demonstrate the mechanism of resistance by Bcl-2 overexpression in this mouse model. We cannot exclude the possibility that the compounds tested herein modulate the cytotoxicity of Dxr through other mechanisms, such as affecting serum half-life of Dxr, increasing intracellular drug levels by altering the expression of polypeptide(s) involved in transport, or by affecting detoxification [49].

Why would inhibiting translation elongation achieve a therapeutic index, since it is expected that translation of all mRNAs would be inhibited to the same extent by such inhibitors? It is possible that, by having higher translation rates [4], [65] transformed cells are more sensitive to these compounds even when they exert partial inhibitory activity. Alternatively, as the cells transform into cancer cells, they are thought to select for higher oncogene activity or expression and become “addicted” to these oncogenes [66]. Some of these oncogenic proteins possess short half-lives and are likely to be selectively depleted by elongation inhibitors driving the transformed cells towards apoptosis. Our work provides a rationale for using elongation inhibitors to modulate chemosensitivity in tumors.

## Supporting Information

Figure S1 Results summarizing in vivo screening data from the NIH Developmental Therapeutics Program for translation inhibitors in various mouse cancer models. The data for each compound was obtained from http://dtp.nci.nih.gov/dtpstandard/dwindex/index.jsp and manually inspected. A positive response in a given model was noted if the Treated/Control cohorts showed a value greater than 125% for any of the given doses, administration routes, or delivery vehicles. The height of the bar graph denotes the total number of different cancer models reported and the open portion of the bar denotes the number of models in which the indicated compound showed activity at least once.
(2.07 MB TIF)

Figure S2 Translation elongation inhibitors potentiate the activity of Dxr to extend overall survival of mice bearing *Pten+/−Eμ-Myc* lymphomas. Kaplan-Meier curves representing the overall survival of mice bearing *Pten+/−Eμ-Myc* tumors following treatment. Ten animals were treated in each cohort. All mice were treated at the same time and in the same experiment, but the data is presented as two curves for ease of visualization. P<0.001 for significance among all curves of combination relative to single agent treatments, as determined by the log rank test.
(1.20 MB TIF)

Figure S3 Overall survival in mice bearing *Eμ-Myc/eIF4E* or *Eμ-Myc/BCL2* lymphomas treated with translation elongation inhibitors. A. Kaplan-Meier curves representing the overall survival of mice bearing *Eμ-Myc/eIF4E* tumors following treatment. Ten animals were treated in each cohort. All mice were treated at the same time and in the same experiment, but the data is presented as two curves for ease of visualization. P<0.001 for significance among all curves of combination relative to single agent treatments, as determined by the log rank test. B. Kaplan-Meier curves representing the overall survival of mice bearing *Eμ-Myc/BCL2* tumors following treatment. Ten animals were treated in each cohort. All mice were treated at the same time and in the same experiment, but the data is presented as two curves for ease of visualization. Log rank analysis of the treatment responses indicates a significant difference between Dxr and Rap+Dxr having a P-value<0.001. The analysis also indicates that the curve obtained with Dxr alone is not significantly different than the ones obtained with HHT+Dxr, Did+Dxr, Bru+Dxr or CHX+Dxr with respective P-values of 0.0149, 0.0241, 0.245 and 0.101.
(1.83 MB TIF)

Figure S4 HHT and Bru trap 80S complexes on mRNA templates. Rabbit reticulocyte lysates were preincubated without compound, with 0.6 mM CHX, 10 µM HHT+0.6 mM CHX or 10 µM Bru+0.6 mM CHX at 30°C for 5 min. The reactions were then supplemented with [^32^P]-radiolabeled CAT mRNA and incubated for an additional 10 min at 30°C. 80S complexes were resolved by centrifugation through 10–30% glycerol gradients. The direction of the arrow indicates the orientation of the gradient, from top to bottom. The total counts recovered from each gradient and the percent mRNA bound in 80S complexes were: CHX (left panel) [26,960 cpm, 20.0% binding], HHT+CHX [28,383 cpm, 16.8% binding], no compound [5744 cpm, 3.4% binding], CHX (right panel) [71,727 cpm, 17.4% binding], and Bru+CHX [50,392 cpm, 12.2% binding] and no compound [11,542 cpm, 2.8% binding].
(1.29 MB TIF)

Figure S5 Translation elongation inhibitors potentiate the activity of Dxr to extend overall survival of mice bearing *Tsc2+/−Eμ-Myc* lymphomas. A. Kaplan-Meier curves representing the tumor-free period in mice bearing *Tsc2+/−Eμ-Myc* tumors following treatment. Ten animals were treated in each cohort. P<0.001 for significance among all curves of combination relative to single agent treatments, as determined by the log rank test. B. Western blot analysis of Rap treatments of mice bearing *Eμ-Myc* (lanes 1 to 2), *Pten+/−Eμ-Myc* (lanes 3 to 4) or *Tsc2+/−Eμ-Myc* (lanes 5 to 6) tumors. Mice were treated for 4 hours with 4 mg/kg of Rap, the tumors extracted and cell lysates prepared and analysed for pan- and p-S6 levels. C. HHT blocks protein synthesis in *Tsc2+/−Eμ-Myc* lymphomas in vivo. Mice bearing *Tsc2+/−Eμ-Myc* lymphomas were treated and polysomes analyzed as described in the legend to Figure 4. These experiments were performed for a total of three replicates with similar results.
(1.64 MB TIF)

Figure S6 Potency of the elongation inhibitors at inhibiting translation in *Tsc2+/−Eμ-Myc* lymphomas. Two hundred and fifty thousand cells were plated in 24-well plates in BCM in presence of increasing concentrations of HHT, Bru, Did B or CHX and incubated for 3 hours followed by a ^35^S-methionine labelling performed 20 minutes before the end of the incubation. The results are expressed as cpm/ug of total protein relative to DMSO control (n = 3).
(1.55 MB TIF)
