# Supplementary figures and images for: Altering Chemosensitivity by Modulating Translation Elongation
Source: PLoS One. 2009 May 1;4(5):e5428. doi: 10.1371/journal.pone.0005428 (PMC2671598; doi:10.1371/journal.pone.0005428)

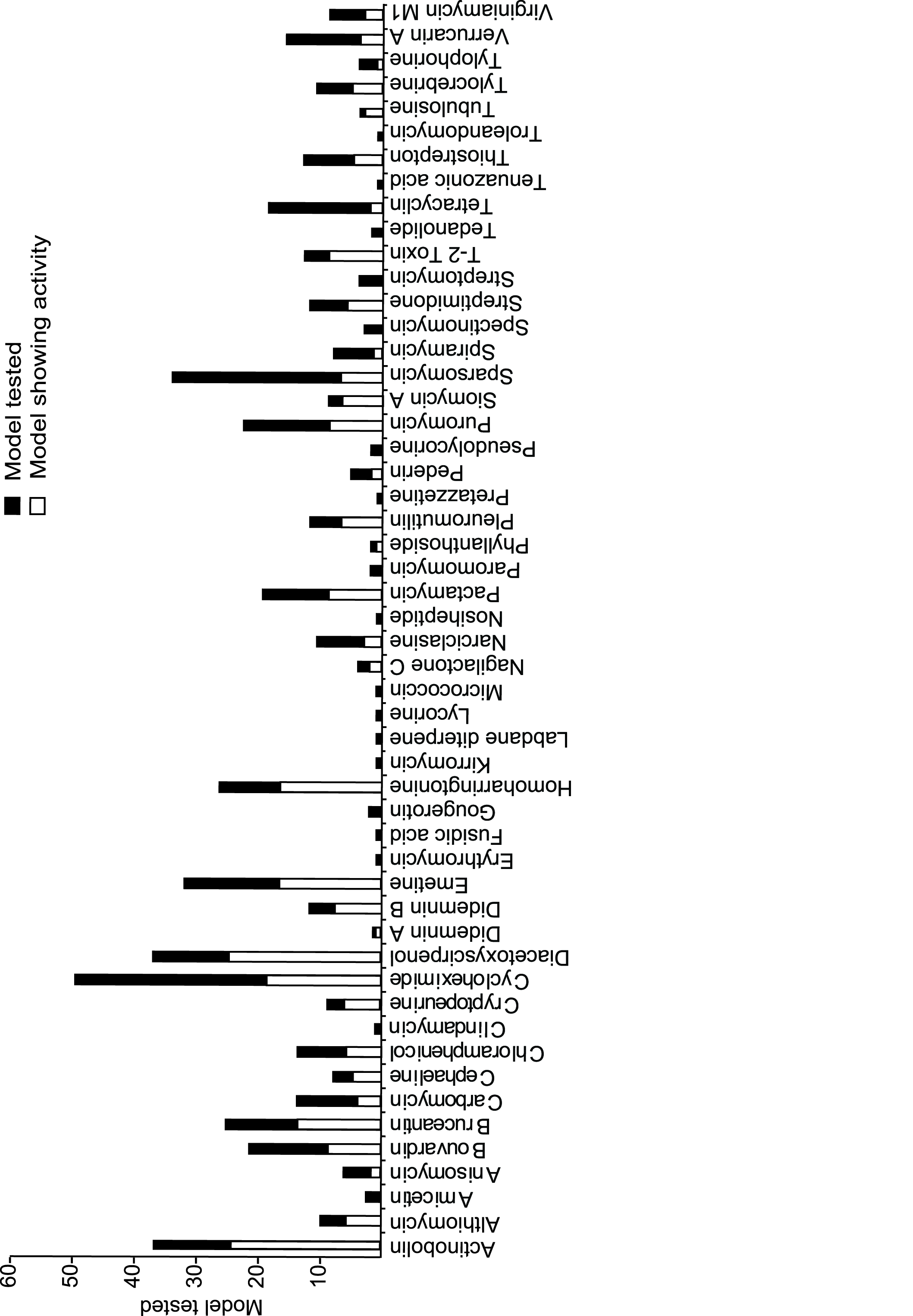

Supplement: Figure S1 — Results summarizing in vivo screening data from the NIH Developmental Therapeutics Program for translation inhibitors in various mouse cancer models. The data for each compound was obtained from http://dtp.nci.nih.gov/dtpstandard/dwindex/index.jsp and manually inspected. A positive response in a given model was noted if the Treated/Control cohorts showed a value greater than 125% for any of the given doses, administration routes, or delivery vehicles. The height of the bar graph denotes the total number of different cancer models reported and the open portion of the bar denotes the number of models in which the indicated compound showed activity at least once. (2.07 MB TIF) [file pone.0005428.s001.tif]

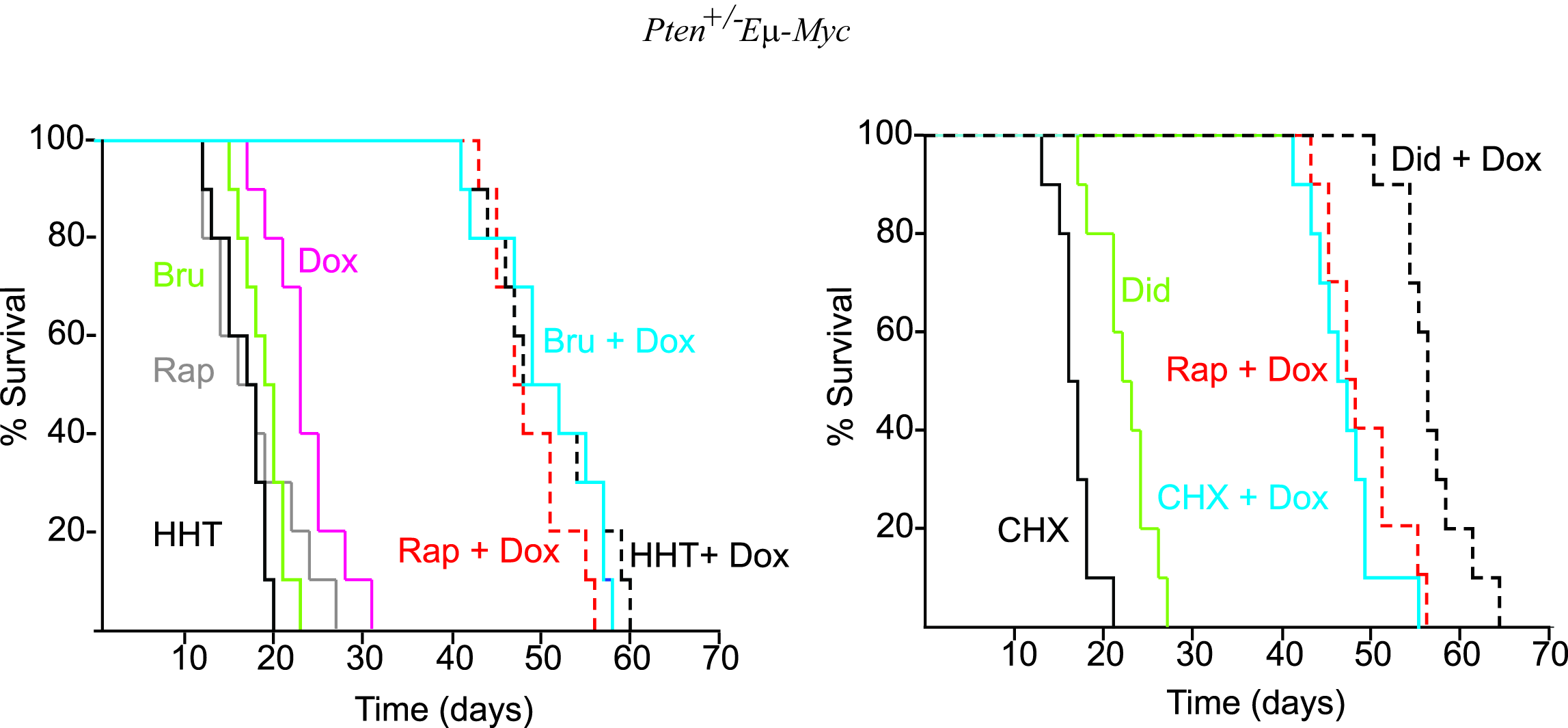

Supplement: Figure S2 — Translation elongation inhibitors potentiate the activity of Dxr to extend overall survival of mice bearing Pten+/−Eμ-Myc lymphomas. Kaplan-Meier curves representing the overall survival of mice bearing Pten+/−Eμ-Myc tumors following treatment. Ten animals were treated in each cohort. All mice were treated at the same time and in the same experiment, but the data is presented as two curves for ease of visualization. P<0.001 for significance among all curves of combination relative to single agent treatments, as determined by the log rank test. (1.20 MB TIF) [file pone.0005428.s002.tif]

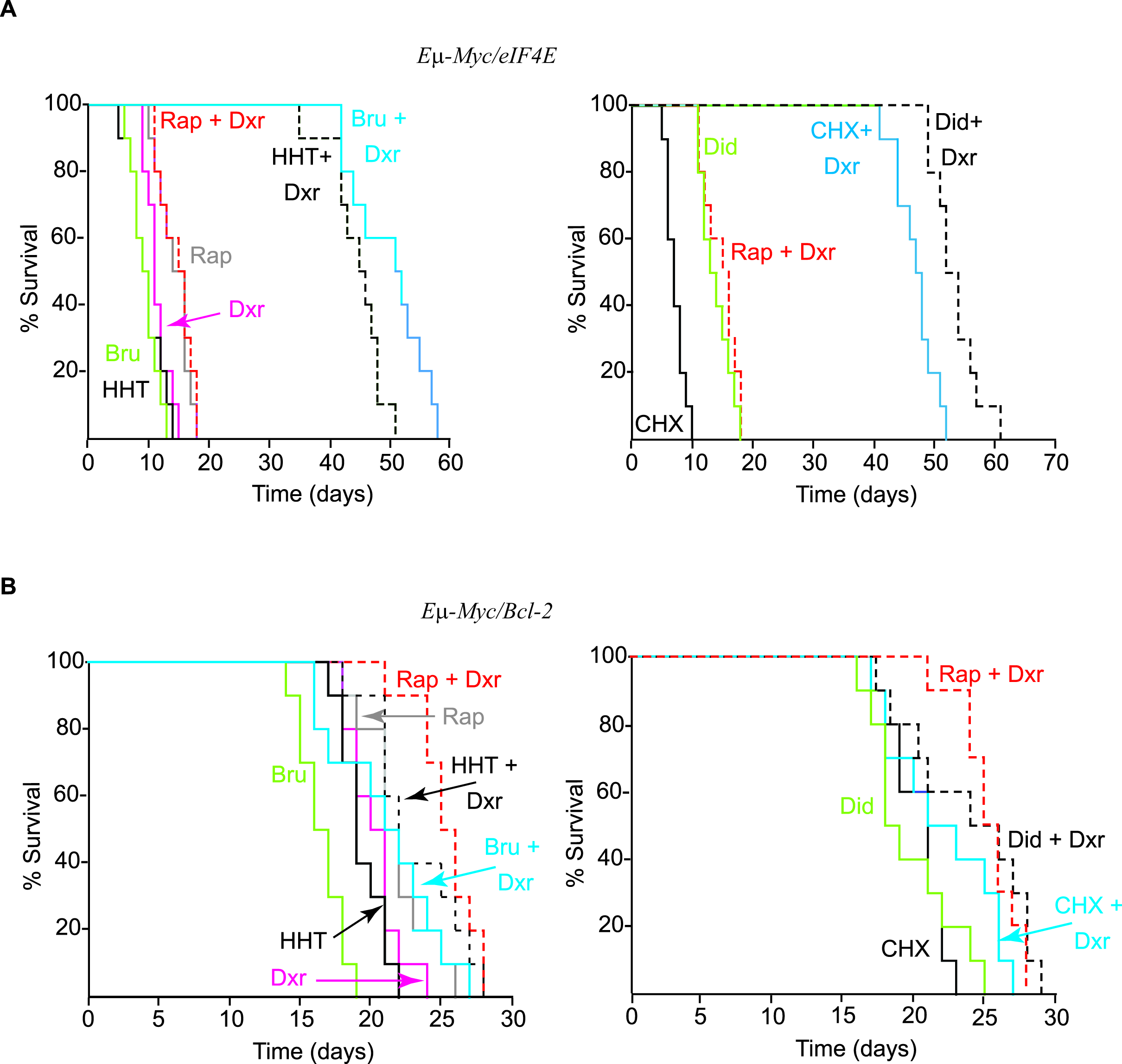

Supplement: Figure S3 — Overall survival in mice bearing Eμ-Myc/eIF4E or Eμ-Myc/BCL2 lymphomas treated with translation elongation inhibitors. A. Kaplan-Meier curves representing the overall survival of mice bearing Eμ-Myc/eIF4E tumors following treatment. Ten animals were treated in each cohort. All mice were treated at the same time and in the same experiment, but the data is presented as two curves for ease of visualization. P<0.001 for significance among all curves of combination relative to single agent treatments, as determined by the log rank test. B. Kaplan-Meier curves representing the overall survival of mice bearing Eμ-Myc/BCL2 tumors following treatment. Ten animals were treated in each cohort. All mice were treated at the same time and in the same experiment, but the data is presented as two curves for ease of visualization. Log rank analysis of the treatment responses indicates a significant difference between Dxr and Rap+Dxr having a P-value<0.001. The analysis also indicates that the curve obtained with Dxr alone is not significantly different than the ones obtained with HHT+Dxr, Did+Dxr, Bru+Dxr or CHX+Dxr with respective P-values of 0.0149, 0.0241, 0.245 and 0.101. (1.83 MB TIF) [file pone.0005428.s003.tif]

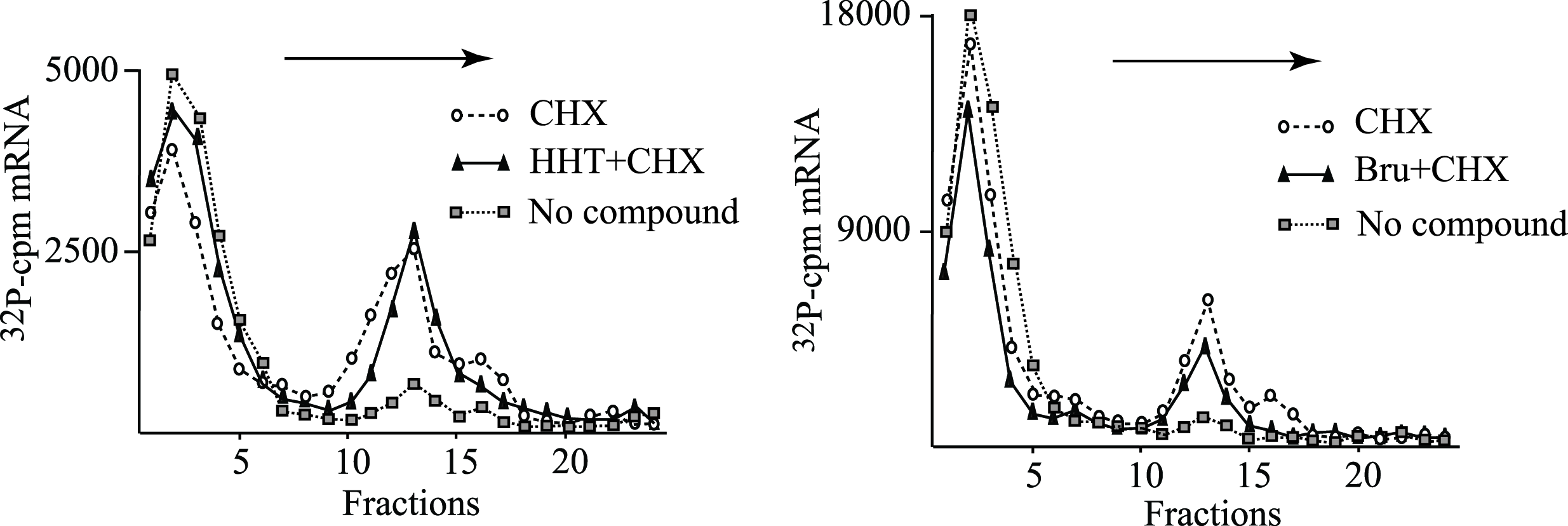

Supplement: Figure S4 — HHT and Bru trap 80S complexes on mRNA templates. Rabbit reticulocyte lysates were preincubated without compound, with 0.6 mM CHX, 10 µM HHT+0.6 mM CHX or 10 µM Bru+0.6 mM CHX at 30°C for 5 min. The reactions were then supplemented with [32P]-radiolabeled CAT mRNA and incubated for an additional 10 min at 30°C. 80S complexes were resolved by centrifugation through 10–30% glycerol gradients. The direction of the arrow indicates the orientation of the gradient, from top to bottom. The total counts recovered from each gradient and the percent mRNA bound in 80S complexes were: CHX (left panel) [26,960 cpm, 20.0% binding], HHT+CHX [28,383 cpm, 16.8% binding], no compound [5744 cpm, 3.4% binding], CHX (right panel) [71,727 cpm, 17.4% binding], and Bru+CHX [50,392 cpm, 12.2% binding] and no compound [11,542 cpm, 2.8% binding]. (1.29 MB TIF) [file pone.0005428.s004.tif]

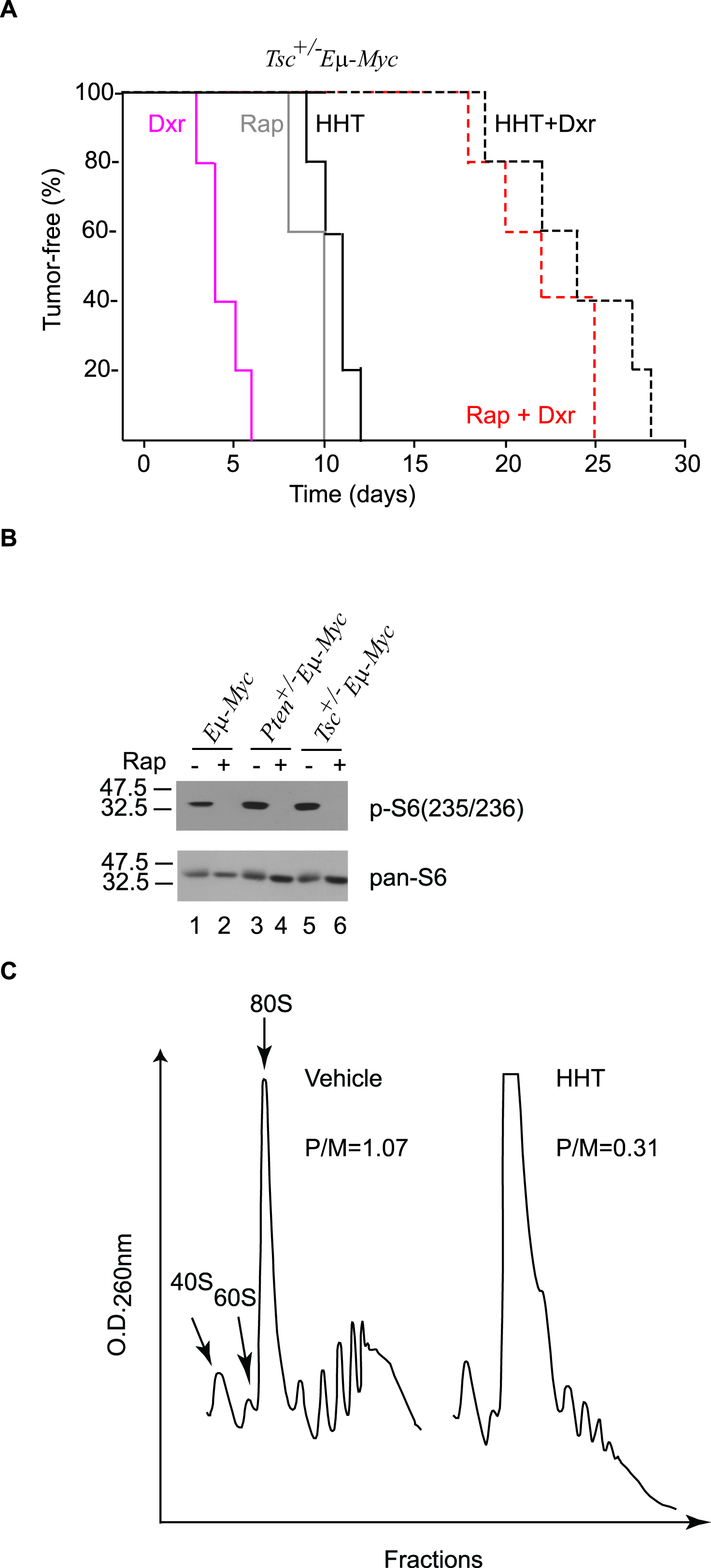

Supplement: Figure S5 — Translation elongation inhibitors potentiate the activity of Dxr to extend overall survival of mice bearing Tsc2+/−Eμ-Myc lymphomas. A. Kaplan-Meier curves representing the tumor-free period in mice bearing Tsc2+/−Eμ-Myc tumors following treatment. Ten animals were treated in each cohort. P<0.001 for significance among all curves of combination relative to single agent treatments, as determined by the log rank test. B. Western blot analysis of Rap treatments of mice bearing Eμ-Myc (lanes 1 to 2), Pten+/−Eμ-Myc (lanes 3 to 4) or Tsc2+/−Eμ-Myc (lanes 5 to 6) tumors. Mice were treated for 4 hours with 4 mg/kg of Rap, the tumors extracted and cell lysates prepared and analysed for pan- and p-S6 levels. C. HHT blocks protein synthesis in Tsc2+/−Eμ-Myc lymphomas in vivo. Mice bearing Tsc2+/−Eμ-Myc lymphomas were treated and polysomes analyzed as described in the legend to Figure 4. These experiments were performed for a total of three replicates with similar results. (1.64 MB TIF) [file pone.0005428.s005.tif]

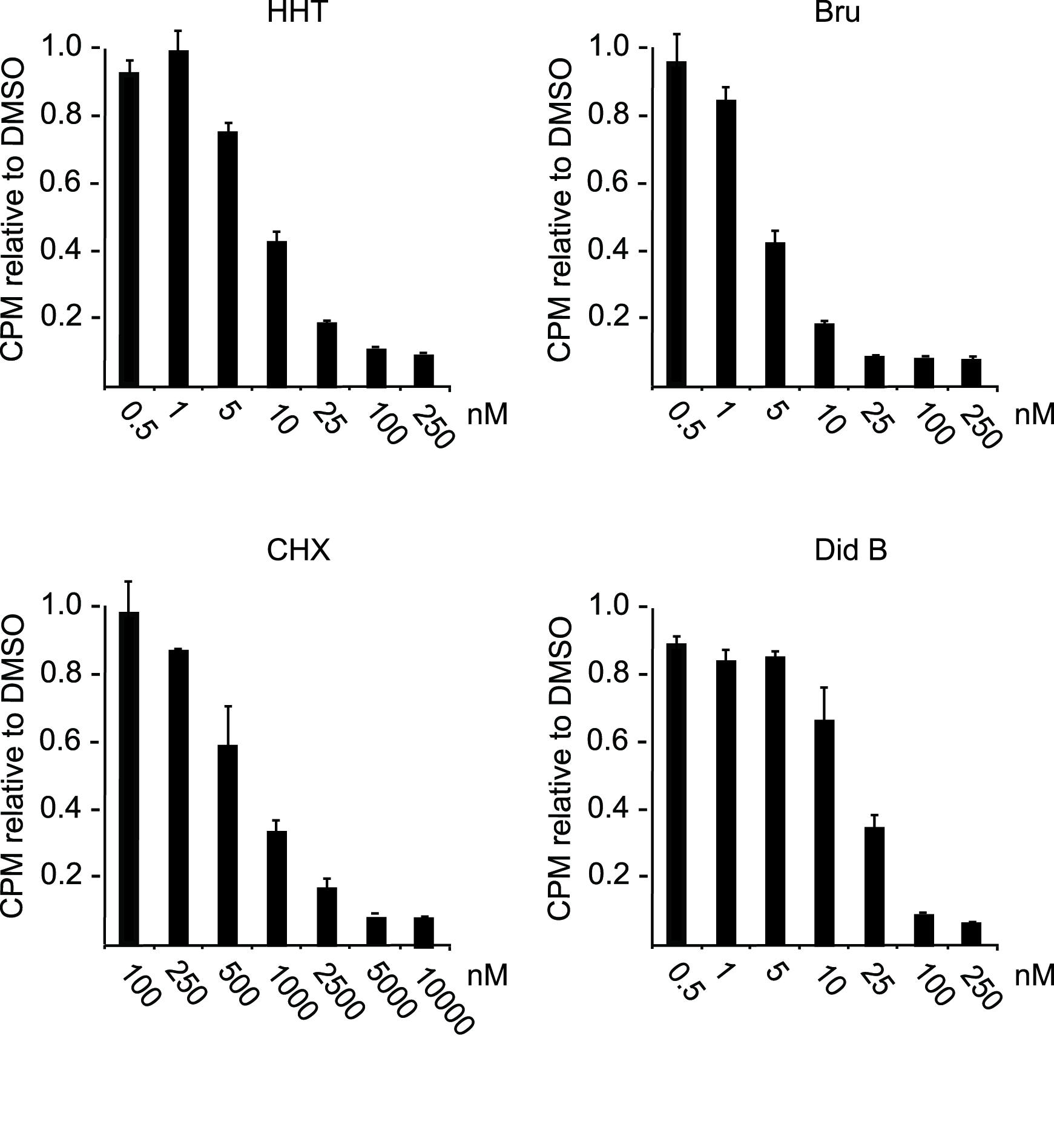

Supplement: Figure S6 — Potency of the elongation inhibitors at inhibiting translation in Tsc2+/−Eμ-Myc lymphomas. Two hundred and fifty thousand cells were plated in 24-well plates in BCM in presence of increasing concentrations of HHT, Bru, Did B or CHX and incubated for 3 hours followed by a 35S-methionine labelling performed 20 minutes before the end of the incubation. The results are expressed as cpm/ug of total protein relative to DMSO control (n = 3). (1.55 MB TIF) [file pone.0005428.s006.tif]
